# Collectanea Medica, Consisting of Anecdotes, Facts, Extracts, Illustrations, Queries, Suggestions, &c.

**Published:** 1818-03

**Authors:** 


### COLLECTANEA MEDICA,

CONSISTING OF

### ANECDOTES, FACTS, EXTRACTS, ILLUSTRATIONS, QUERIES, SUGGESTIONS, &c.

RELATING TO THE

*History or the Art of Medicine, and the Auxiliary Sciences.*

Quicquid agunt medici, nostri farrago libelli.

### *On Diseases of the Skin and Genitals.*

FOR some time past all diseases have been derived from one common source, and only one remedy has been thought necessary for the cure of all. Now, a disease, which for ages has been supposed only curable by that remedy,—a disease, at one time the most intractable,—a morbid poison, against which it was thought the constitution had no powers, is said to be curable, not only without mercury, but almost by the unassisting powers of the constitution. As these discordant opinions rest on authorities not to be undervalued, we shall devote this department of our Number to miscellaneous papers on that subject. Our young readers will pardon us for offering, first, a translation of CELSUS. They may refer, if they please, to the original, but such does not seem to be the practice of their seniors.

### " *Of the Diseases of the Private Parts.*

" The next disorders we are to treat of, are those of the private parts. The names of which, amongst the Greeks, are both more tolerable, and already established by custom; since they are of common use in almost every book and discourse of physicians: with us, the terms are more indelicate, and have had no sanction from the conversation of modest men to qualify their coarseness. This makes it difficult to treat of them so as at once to preserve a delicacy of expression, and deliver plainly the precepts of the art. Nevertheless this circumstance ought not to deter me from writing. In the first place, because it is my intention to comprehend every thing that I have learned to be useful; in the next place, because every body should know how to cure those disorders which we are so unwilling to expose to another.

" Therefore, if the penis be swelled from an inflammation, and the prepuce cannot either be drawn back, or brought forward again, the part must be fomented plentifully with warm water. And, when the glans is covered, warm water must also be injected by a syringe betwixt it and the skin. If the skin, mollified and extenuated by this means, can be drawn back, the subsequent part of the cure is more easy; if the swelling prevails against this remedy, lentils or horehound, or olive leaves boiled in wine, must be applied, and to any of these, while it is rubbed, a little honey is added; and the penis is to be tied up to the belly, which is necessary in the cure of all its disorders; and the patient ought to confine himself to a strict regimen, and abstain from eating, and relieve his thirst by nothing but water. The day following, the fomentation of water must be applied in the same way, and trial made even with some degree of violence to pull back the prepuce; if it will not give way, the surface of it must be slightly cut with a knife. For, when the sanies is discharged, the part will be extenuated, and the prepuce more easily drawn back.

"Whether it has yielded to the latter method, or has never resisted, ulcers will be found, either in the inner part of the prepuce, or in the glans, or in the penis beyond that; which must of necessity be either clean and dry, or humid and purulent. If they be dry, they must be first fomented with hot water; then lycium with wine applied, or oil-lees boiled with the same, or rose-oil with butter. If there is a thin humour in them, they must be washed with wine; then a little honey and oil of roses with butter, and a fourth part of turpentine resin must be used. But, if pus is discharged from them, first of all they ought to be washed with warm mulse; after which is applied, of pepper p. i., myrrh p. ii., saffron, calcined misy, each p. ii. which are boiled with a rough wine to the consistence of honey. The same composition is also proper for the tonsils, a moist uvula, and ulcers of the nostrils and mouth. Another for the same purpose: of pepper p. i. myrrh p. i. saffron p. ii. misy p. i. calcined copper p. ii., which are first rubbed down with a rough wine, then, when they have grown dry, they are again rubbed with three cyathi of passum, and boiled to the consistence of viscum.

"If the ulcer increase in breadth and deepness, it ought to be washed in the same manner; and, either verdigrease, or omphacium with honey, applied; or Andron's composition; or of horehound, myrrh, saffron, scissile allum calcined, dry rose leaves, galls, each p. i., Sinopian minium, p. ii., which are first powdered separately, then mixed and rubbed together in honey, till they acquire the consistence of liquid cerate; after which, they are boiled in a copper vessel gently, so as not to boil over; when the drops of it grow hard, the vessel is taken off the fire; and this medicine, as occasion requires, is softened either with honey or wine. The same composition, by itself, is also good for fistulas.

"Sometimes, too, the ulcer penetrates to the nerves; and there is a plentiful discharge of humour, and thin sanies and fetid, of no consistence, but like water in which recent flesh has been washed; there are pains and prickings in the parts. Although this be of the purulent kind, yet it must be cured by mild medicines; such as the tetrapharmacum plaster liquified with rose-oil, and mixed with a little frankincense; or that which is made of butter, rose-oil,

5

resin, and honey, already mentioned. Above all, this ulcer is to be fomented with abundance of warm water, and covered, and not exposed to the cold.

"Sometimes also, by these ulcers, the penis under the skin is so consumed, that the glans fall off. In which case, the prepuce itself must be taken off by circumcision; and it is a general rule, whenever the glans, or any part of the penis, fall off, or is cut off, that the skin be kept from falling in contact and uniting with the ulcer, so that it cannot be drawn back afterwards, and, perhaps, may even obstruct the urinary passage.

"Tubercles, likewise, which the Greeks call phymata, arise about the glans; which are cauterized, either by medicines, or the actual cautery; and, when the sloughs cast off, copper scales are sprinkled upon them, to prevent any thing growing there again.

"In what we have hitherto described, there is still no gangrene; to which, as in the other parts, so more especially here, ulcers are liable. It begins with a blackness. If this seizes the prepuce, a probe must be immediately put under it, and an incision made; then the extremities are to be laid hold of with a vulsella, and whatever is corrupted must be cut away, and even some of the sound part taken off, and the place cauterized. Whenever any part is burned, the next step is to apply lentils; afterwards, when the sloughs have cast off, the cure is the same with that of common ulcers.

"But, if a gangrene has seized the penis itself, some of the escharotic medicines must be sprinkled upon it, chiefly that which is composed of lime, chalcitis, and orpiment. If medicines fail of success, here, also, whatever is corrupted is to be cut out with a knife, in such a manner as that some of the sound part be taken with it. This rule is as universal as the former, when a gangrened part is cut away, that the wound must be cauterized. But if, either by means of medicines, or the actual cautery, the sloughs have grown callous, there is great danger, that, when they cast off, a profusion of blood from the penis may follow. Therefore, long rest is necessary, and keeping the body almost immoveable, till the sloughs be gently loosened from it in proper time. But, if a person, either wittingly or inadvertently, by walking too soon, has separated the sloughs, and there ensues a hæmorrhage, cold water must be applied. If that does not prevail, recourse must be had to those medicines which stop blood. If even these do not relieve, the part must be cauterized carefully and cautiously; and not afterwards exposed to the same danger by any motion whatsoever.

"Sometimes, also, in the same place, there happens that kind of gangrene, which the Greeks call phagedæna; in which no time is to be lost, but the same remedies must be immediately applied; and, if these are not successful, it must be burnt by the actual cautery. There is, likewise, a certain blackness, which gives no pain, but spreads, and, if we do not resist it, makes its way to the bladder, and cannot be cured afterwards. But, if it be at the end of the glans near the urinary pipe, a small probe should be introduced into that first, to prevent its closing; and then the actual cautery must be applied to the ulcer. But, if it has penetrated deep, whatever is tainted must be cut off. For the rest, it must have the same treatment with other gangrenes.

"There is, likewise, sometimes a callous excrescence from the penis, which is almost void of all sensation, and ought to be cut out. A carbuncle, as soon as it appears there, must be washed by means of a syringe; then it must be cauterized too with medicines, particularly chalcitis with honey, or verdigrease with boiled honey, or sheep's dung, toasted and powdered, with honey. When it falls off, liquid medicines must be used, which are composed for the lips of ulcers."—*Grieve's Translation of Celsus,* p. 336.

The next extract is from Astruc's Collection, and contains the case of Sir Ulrich Hutten, who at last was cured by guaiacum.

Ulrich was no physician, but, by the account he gives of his own case, "he contracted this disease about the year 1509 or 1510, when he was about twenty-one or twenty-two years of age, whilst a soldier in Italy: since he says himself therein, that he had been afflicted therewith for nine years past, that year; and, in that interval, had tried the mercurial illinition eleven times; insomuch, that it was incredible the torture he had undergone from the surgeon's hands, or what sufferings, through the ignorance of physicians."

However, by a subsequent extract from the celebrated Erasmus, it would appear, that Hutten died of the complaint at an early age. Erasmus, who collected the philosophical news of all Europe, describes the disease in the following words:—

"What plague has ever over-run all parts, not only of Europe, but Africa and Asia, with the like celerity as this? What so suddenly penetrates all parts of men's bodies? What so stubbornly resists all helps from art? What so quick in spreading its contagion, or productive of so cruel torment? The vitiligo, besides its deforming the skin, has nothing in it hurtful, and admits of cure: the lichenes with a filthy scurf, disfigure first of all the face, and then the other parts, but still without pain or danger of life, although incurable, unless death should be preferred. But this sickness, besides all that is abhorred in others, carries along with it grievous pain and torment, together with its filth, contagion, danger, and difficulty of cure, by as foul a method; and, after that, is liable to a relapse, as the gout."

Hutten is afterwards supposed to be introduced as exclaiming:—

"This elated and proud scab, although it could never yet obtain a proper name of its own, scorns, notwithstanding, to give precedence to the leprosy, the elephantiasis, the lichenes, the podagra, or the mentagra, in their contention for mastership."

A humorous dialogue follows, which we may hereafter introduce. This case of Hutten's gave rise to Boerhave's Preface which precedes his Aloisinian Collection *de Morbo Gallico.* This justly-celebrated writer was induced to suppose, that the art of treating the disease might have been better known before than in his days; and his confession, that he was unable to prevent secondary symptoms, does him honour, however erroneous his theory may be.

We shall now refer to a few papers, in which diseases are spoken of as having some analogy to, but admitted to differ from, the venereal. Here we meet, not indeed with so long a word as *pseudo-syphilis,* nor so *hellenistic* a combination as *cachexia syphiloidea,* but *L. Ven. notha vel spuria* may do quite as well. The following paper is from a Collection of Essays by Dr. Blair, published by W. Mears,—London, 1718.

*Extract of a Letter to Dr. Mead.*—"The third distemper is what I suppose you have got no account of, being as yet but little known in this country (where it took its rise), except in a few corners thereof. We generally compute its date to commence at the famous battle of Killy-chrankie, soon after which it began to appear. It has not as yet spread far, but, wherever it takes, it is very infectious. It is a commonly received opinion, that the *lues venerea* is not any ways propagated but by impure *concubitus,* and yet this disease is the very same with it, except its not being introduced by a *coïtus,* nor preceded by a *gonorrhœa,* neither for the most part accompanied with *buboes* nor *chankers.* It spares none, affects the sucklings, violently seizes those under age, and such as can be no ways suspected to have a *lues venerea.* It is generally known four ways: first, by nocturnal pains, which degenerate into nodes; secondly, by an exulceration of the throat, landing in a carious palate, and falling off the nose; thirdly, *cacoëthes* ulcers over all the body; fourthly, *cotylidones* here and there upon the body, or dry elevated pustules unequal in the surface, not unlike the fruit of the *rubus idæus,* commonly called with us the *sivvans* (from which the disease takes its name), being much of the same red fleshy colour with that fruit when ripe. The *cotylidones* affect most parts of the body, except the pudenda, in both sexes, by which alone it may be distinguished from the *lues venerea.* This distemper is so contagious, that if any servant labouring under it (as it is generally that rank of people that are most affected with it) do take care of children; be in more frequent converse with other servants; or, in a word, if their bed-cloaths be mixed with those of the rest of the family,—it seldom goes on without communicating itself to some one or other of them. This I have known by experience, and generally those have been either sucklings or underlings, as I have said, about ten or twelve years of age, and sometimes those of betwixt twenty and thirty, whose behaviour could give no ground to suspect its being venereal.

"It now remains, that I enquire into the origin, and inform you of the ordinary cure of this distemper. As to the first, after a more serious consideration of the matter, I can find nothing more reasonable than that after the battle of Killy-chrankie, when the soldiers, being dispersed all over our highlands, had a greater occasion of diffusing their impurities in those parts of the country than perhaps ever before, and of infecting the females there. Now highland nurses being always much valued by those in the lowlands, because of the wholsomeness of their food, had in all probability communicated it to the children on their breasts, upon whom it appeared with that variety of symptoms I have told you of. Again, those children, it seems, by the effluvia or transpirations from their body, communicated it to their dry-nurses, as we call those who take care of them after weaning; and hence it is probable it might have become contagious. This I only give you as a conjecture. However, by the symptoms given, I am of opinion it may be termed *lues venerea notha ceu spuria*, as will further appear by the cure. When first this distemper began to break out, it was generally taken for an inveterate pox, to which no *gonorrhœa* had preceded; whereupon, they endeavoured to carry it off with doses of merc. dulc., which in some succeeded pretty well, in others not at all: for such as are seized with it are most uneasy to work upon; so that I have been obliged to give prodigious doses of mercury without any sensible effect: and, if once mercury be essayed in this distemper, and not brought up to a sufficient height, the patient's body becomes almost impregnable; insomuch, that I have given two scruples of turpeth. mineral for a dose, without any apparent effect; and a solution of mercur. crud. in the spt. of nitre externally to the ulcers, which yet have not yielded. The usual cure of this distemper is strong emetics, either with turpeth. mineral, or gut. gamb.; strong mercurial unctions in case of nodes and nocturnal pains; mercurial lotions and unctions externally to the ulcers, and mercur. dulc. or turpeth. internally, with several purgative doses, alternately given, when we have a mind not to carry it off with a total flux. But, to be sure, this mercurial diet is not to be given over till the cure be performed, otherways the distemper recurs, and becomes more violent, and remedies ineffectual, as is said: so that you see how near of kin this is to the *lues venerea* in its signs, symptoms, and cure.

"It is very observable, that, although this distemper has been raging among us these twenty-four years past, yet it has not much exceeded the bounds of four or five parishes to this hour; so favourable is good Providence in restraining the progress of a distemper which otherwise might prove very noxious to a whole country, and in saving the better rank of people from its infection. I have of late been informed, that it now rages near to Inverness and Inverlochy, which must have been communicated to the inhabitants of those highland countries by the soldiers, as is above observed."

After this, we are obliged to refer to papers in which less attention is paid to the character of local appearances; and diseases are called venereal, merely because they appear on what Celsus, two thousand years ago, called *partes obscœnæ*. We refer to Dr. Michaelis' cure with opium. It is worth remarking, that this physician, notwithstanding the celebrity of his name, has been equally unsuccessful in distinguishing the diseases of the throat, as those of the more usual seat of venereal maladies. Our extract is from the first volume of Medical Communications;—Johnson, London, 1784.

"The use of opium as a specific, in the veneral disease, is one of those new ideas which we owe entirely to accident. No one (so far as I know) ever gave this medicine in venereal cases, unless merely to alleviate pain or lessen irritability, before the year 1779; when the idea of its possessing peculiar antivenereal powers was suggested by the astonishing recovery of a young gentleman, who, after having tried a variety of mercurial preparations, for a most inveterate venereal complaint, gave up his business in New-York, where he then resided, and went to England, without hopes of recovery. He there took large quantities of opium to alleviate his excruciating pains; its effect, however, surpassed his most sanguine expectations, for, without using any other remedy, he found himself perfectly cured within a very short space of time.

"I no sooner heard of this extraordinary recovery, than I became desirous to ascertain whether opium would have the same effect where no mercury had been used. My trials for this purpose have been numerous, and in general attended with great success. They were made with all the accuracy I was capable of. The medicines were always given by the mates of the hospital, and I myself saw the patients daily, and wrote down upon the spot every change that occurred. The patients were so carefully attended to, that I am certain they took the medicines in the doses I directed; and equally certain that they took no mercury or other remedy.

"I think it necessary to mention this, as many persons are prejudiced against observations made in military hospitals, from a supposition that they cannot be made with sufficient accuracy. But I must beg leave to observe, that, when military hospitals are properly conducted, they afford an opportunity for as much accuracy in experiments, as can possibly be obtained in other hospitals, or in private practice. And, in an enquiry like the present, they have this peculiar advantage, that we are able at any time to get information, from the regimental surgeons, whether the patients have had a relapse after their dismission from the hospital.

"The cases I now have the honour to lay before you are only part of my experiments on this subject, (the whole of them you would, perhaps, think too tedious) but I must observe, that they are by no means select ones, as nothing, I think, has been more prejudicial to medical science than the partial accounts of different remedies already published. I acknowledge, indeed, that opium has not always succeeded; but the proportion of the successful cases, to those in which this remedy alone did not effect a cure, was as three to one; and among the cases in which it failed, there were many in which mercury proved equally ineffectual."

Twenty-one cases are *given*, not *selected*, in all which, opium proved a permanent cure.

Many writers have expressed their astonishment at the extinction of leprosy, as well as at its great prevalence a few centuries ago,

The following extract from White's Natural History of Selborne gives a very satisfactory answer to the first of these questions.

"Some centuries ago, this horrible distemper [leprosy] prevailed all Europe over; and our forefathers were by no means exempt, as appears by the large provision made for objects labouring under this calamity. There was an hospital for female lepers in the diocese of Lincoln, a noble one near Durham, three in London and Southwark, and, perhaps, many more in or near our great towns and cities. Moreover, some crowned heads, and other wealthy and charitable personages, bequeathed large legacies to such poor people as languished under this hopeless infirmity.

"It must, therefore, in these days, be, to a humane and thinking person, a matter of equal wonder and satisfaction, when he contemplates how nearly this pest is eradicated, and observes that a leper now is a rare sight. He will, moreover, when engaged in such a train of thought, naturally enquire for the reason. This happy change, perhaps, may have originated and been continued from the much smaller quantity of salted meat and fish now eaten in these kingdoms; from the use of linen next the skin; from the plenty of better bread; and from the profusion of fruits, roots, legumes, and greens, so common in every family. Three or four centuries ago, before there were any enclosures, sown-grasses, field-turnips, or field-carrots, or hay, all the cattle which had grown fat in summer, and were not killed for winter use, were turned out soon after Michaelmas to shift as they could through the dead months; so that no fresh meat could be had in winter or spring. Hence the marvellous account of the vast stores of salted flesh found in the larder of the eldest Spencer* in the days of Edward the Second, even so late in the spring as the third of May. It was from magazines like these that the turbulent barons supported in idleness their riotous swarms of retainers ready for any disorder or mischief. But agriculture is now arrived at such a pitch of perfection, that our best and fattest meats are killed in the winter; and no man need eat salted flesh, unless he prefers it, that has money to buy fresh.

"One cause of this distemper might be, no doubt, the quantity of wretched fresh and salt fish consumed by the commonalty at all seasons as well as in lent; which our poor now would hardly be persuaded to touch.

"The use of linen changes, shirts or shifts, in the room of sordid and filthy woollen, long worn next the skin, is a matter of neatness comparatively modern; but must prove a great means of preventing cutaneous ails. At this very time woollen instead of linen prevails among the poorer Welch, who are subject to foul eruptions.

"* Viz.—six hundred bacons, eighty carcasses of beef, and six hundred muttons."

"The plenty of good wheaten bread that now is found among all ranks of people in the south, instead of that miserable sort which used in old days to be made of barley or beans, may contribute not a little to the sweetening their blood and correcting their juices; for the inhabitants of mountainous districts, to this day, are still liable to the itch and other cutaneous disorders, from a wretchedness and poverty of diet."

The following extract from the last edition of Adams on Morbid Poisons, is produced to show how recently a most formidable disease has not only been exterminated, but, for want of due records, that the events of only thirty years scarcely exist in the memory of any living person.

"Dr. Swediaur, and, after him, Mr. Bell, mention a disease in Canada, in many respects similar to sivvens. Dr. Swediaur tells us his account is taken from the papers of Governor Hamilton, on whose representation six surgeons were sent from England, to give gratuitous assistance to the poor sufferers.

"It is probable many of the symptoms described were unconnected with the disease, not only because no morbid poison can assume such a variety of forms, but because such appearances often occur from other causes. Among the rest, we meet with mortification of the feet and other joints, which, in the north of Canada, would be more properly suspected to arise from frost-bitten. This inaccuracy is not to be wondered at, as the account was not taken from the papers of a medical man. The only professional name quoted is Dr. Bowman.

"Ten years ago, when the first edition of Morbid Poisons was printed, I made every enquiry after Mr. Bowman (as he is called by Mr. Bell), but his name was unknown to the booksellers and medical literati. On the present occasion my enquiries have been equally unsuccessful among all those mercantile gentlemen who have connexions in Canada, among those public officers concerned for the colonies, and particularly for Canada, and also such medical gentlemen as would be likely to be consulted, before a mission of six surgeons should have been sent from England.

"If such an event really happened so lately as 1786; and if, as Dr. Swediaur observes, in the year 1785, five thousand eight hundred persons in Canada were reported infected, besides those who concealed their complaints, it is scarcely possible that all these enquiries should have been unsuccessful. Indeed, it is evident that there must be some inaccuracy in Dr. Swediaur's account, as no gentleman of the name of Hamilton has ever been governor of Canada. However, there is sufficient internal evidence that some disease, very similar to sivvens, has appeared at the Bay of St. Paul in Canada, and probably from similar causes.

"The friendship of Dr. Pitcairn has favoured me with a manuscript and with the following history. About twenty-five or thirty years ago it was put into his hands, with other papers of natural history, by the Hon. John Cochrane, who had resided several years as a merchant in Canada, and was, I believe, one of the council of the colonial government. It was said to be written by a Mr. Beaumont, a French surgeon, who had been sent from Quebec by Governor Haldiman to investigate the nature of a distemper in the Bay of St. Paul. Dr. Pitcairn copied the MS. which contains the description of a disease in many respects similar to Dr. Swediaur's account, and also to sivvens. In one respect they all agree, that the contagion is often conveyed by smoaking with the same pipe.

"There is, therefore, reason to believe, that such a disease has existed in the Bay of St. Paul in Canada, though probably the account has been greatly exaggerated. Nor is it impossible, when we perceive that Dr. Swediaur writes Hamilton for Haldiman, and Bowman for Beaumont, that the surgeons may have been sent from Quebec instead of London. Here I shall, however, dismiss the subject, having only introduced it to show the difficulty that attends an enquiry into the validity of an event, which appears to be supported by official documents, which must be highly interesting to all, and we should suppose have been submitted to the consideration of those medical men who reside in the metropolis."

The following is extracted from the last Volume of Medico-Chirurgical Transactions, and forms an additional proof how much cutaneous diseases are confounded; how much they depend on poverty and its attendant privations, and also how easy it is to refer all such to the leprosy *of the middle ages,* of which we know so little.

"*On the Pellagra, a Disease prevailing in Lombardy;* by Henry Holland, M.D. F.R.S."

The first paragraph informs us that Dr. Holland had a short residence in the north of Italy at two successive periods. That many of his remarks are founded on his observations at the hospital at Milan and *elsewhere*, and the remainder on conversation and the perusal of two or three of the several treatises on the Pellagra. The second paragraph discusses the *class* or *order* whether it shall be leprosy, scurvy, or ichthyosis. An account of the districts principally invaded by it follows, after which the author remarks:—

"Before alluding to the probable causes which determine this locality and extension of the disease, it will be desirable to relate the symptoms which characterize it in the most ordinary form of its occurrence; though these, I may remark in the outset, display frequent varieties, and are often complicated with other characters of disease. The pellagra is a malady confined almost exclusively to the lower classes of the people, and chiefly to the peasants, and those occupied in the labours of agriculture. Though there are doubtless exceptions to this statement, yet the facts it includes are so general, as to render it of importance in the history of the disease. The first distinct appearance of the malady is under a local cutaneous form; preceded, however, occasionally by languor, debility, and other indications of a general cachectic state of body. The local symptoms very generally shew themselves in the first instance, early in the spring, at the period when the mid-day heat is rapidly increasing, and when the peasants are most actively engaged in their labours in the fields. The patient perceives on the back of his hands, on his feet, and sometimes, but more rarely, on other parts of the body exposed to the sun, certain red spots or blotches; which gradually extend themselves, with a slight elevation of the cuticle, and a shining surface, not unlike that of lepra in its early stage. The colour of this eruption is a somewhat more obscure and dusky red than that of erysipelas: it is attended with no other uneasy sensation than a slight pricking or itching, and some tension in the part. After a short continuance in this state, small tubercles are frequently observed to arise on the inflamed surface; the skin almost always becomes dry and scaly, forming rough patches, which are excoriated and divided by furrows and rhagades. Disquammation gradually takes place, which, though it leaves behind a shining unhealthy surface in the parts affected, yet, in the first year of the disease, is rarely followed by a repetition of the appearances just described. Towards the close of the summer, or occasionally still earlier, the skin has resumed nearly its natural appearance; and but that the further progress of the disease is familiar to every inhabitant of the country, the patient might be led to flatter himself that the evil was gone by, and that there was no particular reason to dread its recurrence.

"As the persons who are affected by the pellagra in this incipient stage, are rarely brought into an hospital, I have had few opportunities of observing the complaint thus early in its progress. From the instances I have seen, however, I should consider the description just given of the local eruption, as being accurate in all material points. With this local affection are connected, even in the first period of the disease, certain general symptoms, which are important in as much as they indicate the constitutional nature of the malady. Debility of the whole body; vague and irregular pains of the trunk and limbs, but especially following the track of the spine and dorsal muscles; headache, with occasional vertigo; irregular appetite, and general depression of spirits; these are the more ordinary symptoms which attend the early part of its progress. The bowels are for the most part relaxed; and usually continue so in the further course of the disease. There are no febrile symptoms; and in females the menstruation is generally continued without irregularity.

"The remission which the patient obtains during the autumn and winter of the first year, is almost universally followed by a recurrence of his symptoms in the ensuing spring, under a more severe form, and with much greater disorder of the constitution. The cutaneous disorder is removed, and spreads itself more extensively; though still affecting chiefly the hands, neck, feet, and other exposed parts of the body. The skin becomes callous and deeply furrowed; and large rhagades shew themselves, especially among the articulations of the fingers. From the cases I have seen of the cutaneous affection in this stage, I should speak of it as most resembling the inveterate degree of psoriasis, or of the lepra vulgaris, with some affinity certainly to the ichthyosis, under which, as I have already mentioned, Alibert has classed it. The debility is greatly increased in the second year of the complaint; frequently depriving the patient of all power of pursuing his active labours, and rendering him peculiarly susceptible of all changes of temperature. Partial sweats frequently break out without any obvious cause. All the nervous symptoms of the first year are renewed in a more severe degree; there is a general tendency to cramp and spasmodic affections; the mind begins to suffer under the disorder; and the feeling of anxiety and despondence is very strongly marked. I find the symptom of *libido inexplebilis* noticed by one or two writers; but I did not learn this as the result of my own inquiries; and I am rather disposed to attribute the opinion to the credulity common upon this topic, or perhaps to a desire of associating the pellagra more closely with the leprosy, as described by ancient writers. The other symptoms already noticed, make progress as the heat of summer advances, and with greatest rapidity in those patients who are much exposed to the sun. As in the preceding year, they begin to decline towards the middle or end of the autumn; but the remission, as well of the local affection, as of the general disorder, is much less complete than before, and the patient continues to suffer during the winter from the debility, and other effects, consequent upon the disease.

"In the third year every symptom is renewed at an earlier period, and in an aggravated degree. The constitutional malady shews itself under a variety of forms; some of the symptoms having considerable analogy to those of scorbutus; all of them indicating a general cachexy of habit, and more particularly a lesion of all the voluntary functions. The debility now becomes extreme: the patient is scarcely able to support himself; and the limbs, besides their feebleness, are affected with pains, which still further impede the power of motion. The diarrhœa continues, and tends of course to augment this debility. Frequently a dysenteric state of the bowels comes on in the latter stages of the disease. The breath is generally fetid; and the odour of the matter perspired often extremely offensive. The appetite and digestion are irregular, yet, on the whole, perhaps less affected than most of the other functions. Dropsical effusions now frequently come on; occasionally ascites, but more commonly anasarca. Vertigo, *tinnitus aurium*, and double vision, are almost universally concomitants of this stage of the disorder, and all the senses become exceedingly impaired. Some spasmodic affections are very general, and these not unfrequently take a very decided epileptic form.

"Connected with these latter symptoms is the effect which the pellagra produces on the minds of those suffering under the malady, which effect forms one of the most striking circumstances in the history of the disease. The anxiety, watchfulness, and moral depression of the patient are rapidly augmented. In the hospitals appropriated to the reception of such cases, the *Pellagrosi* afford a melancholy spectacle of physical and moral suffering, such as I have rarely had occasion to witness elsewhere. These unhappy objects seem under the influence of an invincible despondency; they seek to be alone; scarcely answer the questions put to them; and often shed tears without any obvious cause. Their faculties and senses become alike impaired; and the progress of the disease, where it does not carry them off from debility and exhaustion of the vital powers, generally leaves them incurable idiots, or produces occasionally maniacal affections, which terminate eventually in the same state. As a striking proof of this tendency of the disease, I may mention the fact, that at the time I visited the Lunatic Hospital at Milan, there were very nearly 500 patients of both sexes confined there; of which number, more than one-third were *Pellagrosi*,—people brought thither by the termination of their disorder, either in idiocy or mania. Even this statement gives little adequate idea of the ravages of the disease, in this mode of its termination. The public hospitals of the country are wholly incompetent to receive the vast number of persons affected with the pellagra; and the greater portion of these unfortunate people perish in their own habitations, or linger there a wretched spectacle of fatuity and decay. Where debility, as generally happens, is the cause of death, it manifests itself in the latter stage, with the usual concomitants of colliquative diarrhœa, spasmodic affections, and coma; and produces a degree of emaciation, scarcely to be surpassed in any other disease.

"The mania consequent upon pellagra is often of a very violent kind, of which I have seen some remarkable instances in the Lunatic Hospital at Milan. Where such affections occur, the progress of the disease appears to be in some degree retarded, and the strength less rapidly declines. Attempts at suicide are frequent among Pellagrosi; and particularly, it is said, by throwing themselves into the water. Strambi has thought it worth while to characterise this tendency by the name of Hydromania.

"Though, for the sake of brevity, I have described this train of symptoms as going on from the third year, yet I may remark that the pellagra is generally of longer duration; and that other intermissions usually occur in its progress, giving the patient a certain relief in the degree of his sufferings, though little hope as to the event of the disease. In some instances the cutaneous affection forms the principal indication of the complaint for several successive years; being renewed every spring, and disappearing again in the autumn. In other cases, where it has been found possible to remove the patient to a new situation and mode of life, the disease is still further arrested in its progress. It rarely happens, however, that these means can be practically adopted; and the constitutional malady is generally so far established in the third or fourth year, that little hope remains of benefiting the patient, either by medicine or change in the mode of life."

Some remarks follow on morbid dissections, and on the hereditary nature of the complaint. The first are said to afford little information. As to the second, we know not how a complaint so generally extending among the poor, and arising from causes to which they are all exposed, can be ascertained to be hereditary.

"The question whether the pellagra be contagious or otherwise, has of course attracted the attention of all who have had opportunities of observing the disease. This point, as I believe, on the best authority, has been decidedly settled in the negative. Setting aside particular instances, the general facts may be mentioned, that the disease is almost exclusively confined to the lower classes; that of people living together in the same habitation, some have the disorder, others not; and that, in the case of married people, it frequently happens that the husband or the wife is pellagrous, while the other remains wholly unaffected.

"From the preceding history of pellagra, in its ordinary form of occurrence, it will be seen that the disease has in some points a certain degree of *affinity to the leprosy of the middle and early ages;* and this relation has been noticed by most of the authors who have written upon it. The differences, however, in other points are so distinctly marked, as not to admit of the diseases being classified under the same name; and this remark may be further applied to the scurvy, with which the pellagra is connected by several common symptoms. By some authors, indeed, it has been described under the name of the scorbutus alpinus; but the greater number have considered it (and apparently with more reason) as a separate disease, though occasionally complicated with the genuine scurvy, which prevails as a frequent disorder in the same districts of country. Titius, one of the writers upon the pellagra, has defined it *erysipelas periodicum, nervosum, chronicum;* taking, as the basis of this definition, the appearance of the eruption as it first occurs on the skin. Its after progress, however, and the concomitant constitutional symptoms, are so different from those of erysipelas, in the common understanding of the term, that it would be difficult to admit such a definition, as giving the nosological arrangement of the disease."

The period at which the disease first appeared is differently conjectured by different writers. Among others, there is a doubt started, whether pellagra is not the same as the pellarella of the sixteenth century.—A more important consideration is, a progressive increase in the number of sufferers compared with the inhabitants,—which is said to be from one in twenty to one in five, and to be alarmingly augmenting. This is not improbable, by the accounts which follow on the supposed cause of the disease.

"The immediate effect of these privations is obvious in the aspect of squalid wretchedness and emaciation, which forms so striking a spectacle at the present time throughout the greater part of Lombardy. I say particularly *at the present time,* because, whatever may have been the progress of misery among the peasants of this country during the last half century, it appears to have increased, in a tenfold ratio, during the last two years; the effect of bad harvests, added to the preceding wars and political changes which have distressed this part of Italy.

"This general condition of the peasants of Lombardy seems to be the cause to which we may most reasonable attribute the prevalence of the pellagra amongst them. Their bad and deficient diet may be presumed to produce a cachectic habit of body; manifesting itself progressively in the cutaneous eruption, in muscular debility, and in a general lesion of the nervous system; including the senses, and the mental faculties. I am aware, that I am here using very general phrases; but it will be readily admitted, I believe, that the state of our knowledge, as to the causes of disease, does not often admit of others more definite; and, though I might cite various speculations which the writers on pellagra have adopted, founded chiefly on the doctrines of the humoral pathology, yet I refrain from this, under the conviction that they have added little or nothing to our real knowledge of the subject. The general fact that a deficiency of good nourishment and other means of comfortable subsistence, is capable of producing constitutional disease, is attested by numerous analogous instances. The scurvy may be mentioned as one closely allied in several respects, though differing from the pellagra, as well in its symptoms as in the particular nature of the diet, which appears to have effect in producing the disorder. Other forms of disease, more closely akin to the pellagra, I have observed in Iceland; connected apparently with the diet and mode of life of the inhabitants of that island. Some of these I have described in a Thesis, on the Diseases prevailing among the Icelanders, published in 1811. Here too it may be remarked, that the peculiarity of diet is of different kind, and that the points of similarity of condition are chiefly such as relate to general privation and want of cleanliness; causes which appear to have more effect in producing the diseases of this class, scurvy perhaps not excepted, than any of those peculiarities of animal or vegetable food, which are usually alleged and insisted upon by medical authors."

Though the symptoms may be closely allied to scurvy, yet we are told they have a greater similarity to a disease in Iceland, which is the more remarkable, as it exists in Italy only, or chiefly in the summer months; and is by some imputed to insolation. But "the *leprosy or elephantiasis* of the middle or *dark* ages" is again said to come the nearest to the pellagra. This, we presume, means that we know little or nothing of either. How much more enlightened are we in these times! A number of remarks follow from various writers, quoted, we presume, by the few the author has had an opportunity of perusing. After which, says Dr. Holland:—

"Before concluding this paper, I may notice briefly the existence of a disease in the Asturias, which has been described by Thiery; and which, from its analogy to the pellagra in many of its symptoms, has been alluded to by several of the writers on the latter disease. The description of Thiery, however, would make it appear, that this malady much more closely resembles the *lepra arabum or elephantiasis;* the general relation of which to the pellagra, I have already had occasion to point out. I am not aware of the peculiar circumstances which produce or maintain this disease in the Asturias; but it is not unlikely that they resemble those which have been already mentioned as the probable causes of the pellagra of Lombardy; and which appear capable of producing several different forms of constitutional disease, attended with cutaneous eruptions."

In the opinion of some of our readers, it will now be thought our duty to ascertain the class, order, genus, species, and perhaps the varieties, of pellagra. But, as we have several times confessed our dulness in nosology, we shall avail ourselves of the labours of others. The first, as a criticism, takes a comprehensive view of the question. We shall transcribe the words.

"In our analysis (say these writers) of Mr. Good's Physiological System of Nosology, we doubted the propriety of his placing the pellagra *Italica* under Bucnemia, a genus which has the closest affinity with Elephantiasis; stating, as our reason, that the Italian pellagra is a highly inflammatory affection. This opinion we have lately had an opportunity of examining, by an account of this disease, drawn up from actual observation, by Dr. Holland. From the perusal of that account, we must now admit, that the Italian pellagra is not a disease of that high inflammatory action which we were informed it is: nevertheless, it appears to have less affinity to elephantiasis than to scurvy, although the particular diet producing the pellagra differs very materially from that which is supposed to be the cause of scurvy. The pellagra is apparently hereditary; but Dr. Holland conceives that the physical condition of the peasants of Lombardy is the most probable cause of the prevalence of the disease in that district."

If there be really a *physical* difference between the peasantry and the other classes, or between the peasantry of that and other districts, we should conceive that such difference must be hereditary. If the difference is in food, we recollect, that diet was at one time considered among the *non-naturals;* and, whimsical as we once thought the expression, we now conceive it may serve as a distinction among the causes of diseases, by separating the physical or natural condition of the man from the effects of artificial causes. But we are wandering from nosology. The following account is copied from Mr. Good's Physiological System.

"Elephantiasis Italica. Tubercles chiefly on the body and limbs; sometimes disquammating; great tension of the skin; vertigo; burning, lancinating pain in the head; melancholy; at first remitting, afterwards fixt, terminating in alienation of mind; hereditary.

Pelagra. *Jansen. Frapolli,* Animadv. in morb. vulgo Pelagram, Milan 1771. Della Pelagra descrizione di *Mich. Gherardini,* Mil. 1780. *Thierri,* Recueil périodique d'Observations de Medicine: Paris. 1755.

Pellagra. *Oleggio.*

Tuber Pellagra. *Parr.*

Mal del sole. *Mediolan.*

3. Asturiensis. Tubercles chiefly on the hands and feet; crustaceous, desquammating; continual tremor of the head and upper part of the trunk; baldness of the scalp as well as of other parts: gloom and terror of mind.

Lepra Asturiensis. *Sauv. Thierry.* Journ. de Med. Maie 1755.

Mal de la Rosa. *Spanish.*"

"*Elephantiasis Italica.* For a knowledge of this species of elephantiasis, as the author ventures to denominate it, we are almost exclusively indebted to Italian physicians, by whom it is called pellagra or *pelagra—skin-affection,* from *pellis,* and ἀγρα, a barbarous term, as compounded of two different languages. The first writer upon the disease appears to have been Francis Frapolli, a Milanese physician, whose work 'In Morbum vulgo Pelagram,' &c. was published at Milan in 1771, and who expresses himself doubtful whether the disease, though not antecedently described, is not referred to occasionally by earlier writers, though he does not think that the Pilarella, *as the syphilis was called, when it proved depilatory to the chin and eye-brows,* was the disease in question, notwithstanding this seems to have been an extensive opinion at the time. The next tract, of any note upon the subject, was published at Venice in 1784, by G. M. d'Oleggio, under the title of Tr. Teo. Prat. delle Malattie del insolato di prima vera volgarimente dette della Pellagra. But the best account we have received of the disease is from the pen of Dr. Jansen, of Leyden, which appeared in 1788. Dr. Jansen has entered into a very full inquiry upon every thing that relates to it, and his description is copied, or rather condensed, in the present definition. It is singular that he could not hit upon the origin of the term, and questions whether it may not be derived from the Greek πέλας. He tells us, that it was occasionally called *mal del sole,* as though produced by the heat of the sun. The cause of the disease, after all, seems uncertain; it is found in various parts of Italy; but especially in the Milanese and Venetian districts, so widely differing from each other in soil and situation, that it can scarcely be referred to these sources. Yet it is endemic and hereditary; so that the condition of the peasantry in these regions is truly miserable. Dr. Parr is, perhaps, the only writer who has hitherto introduced this disease into a nosological arrangement."

Mr. Good's account of *Bucnemia* follows, as the next succeeding genus in this fourth order of his third class.

"Leg enormously tumid, and mis-shapen; skin thickened, livid, rugose; often scaly; scrotum, arms, or other parts, sometimes participating in the affection.

Elephantiasis. *Auct. Var.*
Dal fil.
Drüsenkrankheit. *G.*
Yava-skin. *Polynes Isles.*
*Barbadoes-leg. Hillary,* Dis. of Barbad.
*Glandular disease of Barbadoes. Hendy.*"

"Gen. X. Bucnemia, from βου, an augmentive particle, evidently derived from the Hebrew בע or בעה, *(bo* or *boa),* 'to swell, grow large, or intumesca,' and κνήμη, 'crus,' or 'the leg,' literally *bulky or tumid leg.* It is, in truth, the *dal fil.*"—*Good's Nosology,* page 262.

Then follows a confirmation of the jumble and perplexity of dictionaries in the Arabic and connate languages, as was remarked on a former occasion. To do justice to Dr. Parr, we must admit, that in a much smaller space we met with all, and more than is contained in Dr. Holland's paper; which is the most correct we pretend not to know. However, Dr. Parr has greatly the advantage in his reference to authors, which occupies nearly three-fourths of a closely printed column in 4to. The similarity of the accounts renders it unnecessary to offer any long extract, especially as our principal business is with the nosological character.

"Pellagra, (from *pellis, the skin,* and αγρα, *a disease.*) This singular disease has attracted particular attention within these last thirty years, not wholly from its novelty, but from its becoming more violent, and from its being more generally accompanied with peculiar nervous affections. It comes near the *lepra asturiensis* of Sauvages, but seems more closely to resemble the *elephantiasis,* as we have described it, vol. i. p. 598."

In the article alluded to, we find a number of diseases included, which we know not how to discriminate. We, therefore, content ourselves with transcribing the first paragraph.

"Elephanti'asis; *lazari morbus vel malum; Phœniceus morbus,* is generally ranked as a species of leprosy (see Lepra Arabum); but is distinguished from the leprosy by being seated in the *flesh,* while the leprosy only affects the *skin,* or, at most, the integuments. This disorder receives its name from its often affecting the legs, so as to make them resemble those of an elephant; but, in many instances, the legs are not affected."—*Parr's Medical Dictionary.*

We have devoted so much time and space to this subject, in order to relieve some of our younger readers from an apprehension concerning the apparent difficulties of nosology and its supposed necessity. It is far from our wish to encourage them in inattention to this or any thing else connected with medicine. Nosology, the methodical we mean, might have been useful, had it progressively improved since its first introduction into medical science. But, when we reflect on the caution of Cullen,—" *De*

*Elephantiasi, &c. utpote morbis a me nunquam visis amplius statuere non* AUSUS SUM," and perceive that this same Elephantiasis has been since used to classify one disease commencing for the most part in the face, with a disease usually first appearing in the foot, and with a third commencing at a certain period of the year, on the hand; a tubercular disease which emasculates the subject; a glandular disease which, after filling the cellular substance, only enlarges the scales of the cuticle; a disease said to arise from the influence of the sun, from poverty, under privations and precarious security, yet to be hereditary; when it is compared with a disease known only by the uncertain and confused traditions of the *dark or middle* ages; when to all this, we add the various cuticular appearances which it is said to assume,—can we do a kinder office to our young readers, than encourage them to pursue their studies without the terror that must follow an apprehension of the necessity to reconcile, or even to comprehend, such discordant accounts?

It would be too irksome, not to say too painful, a task to collect all the contradictory characters under which this pellagra is confounded. It will be enough to include it in the Elephantiasis or Leprosy of the darker ages,—the only thing in which all or most of the writers seem to agree. We should however remark, that, by some, it is associated with Pelarella, as syphilis was called *when* it "proved depilatory to the chin and eye-brows." We should have been better pleased, had it been said—when S. was considered so: for some time after the appearance of syphilis, all anomalous diseases on the skin, which had before been imputed to leprosy, were consigned to the new disease. Since the time of Mr. Hunter, this has been gradually going out of habit in England: on the continent, as we shall presently see, it continues almost to this day. But first, to relieve the dullness of this long article, we shall extract some humorous passages, one from a professor of medicine, the other a profound scholar,—both the early reformers, one of medicine, the other of religion,—both well acquainted with the literature and philosophy of the age when syphilis is supposed to have made its first appearance. Archibald Pitcairn remarks, "*Lepra ante famam morbi neapolitani Hydrargyro cessit nomenque nunc amisit.*"

Erasmus, with his constitutional drollery, shows us that, if such was the universality of this new disease, it would be in vain to attempt escaping it, meaning, no doubt, that every disease for which no other name could be discovered, was considered syphilitic. The following humorous dialogue is not, we believe, inserted in the school editions of his Colloquies.

"*P.* I would have the men castrated, and the women shall be confined. *G.* This indeed would cut off the brood, and prevent *ne ex malis corvis, nascantur mala ova.*—But you forget, this evil is propagated many ways besides that of copulation, as by kissing, talking together, touching each other, drinking together: for such is the spiteful nature thereof, that it is never better pleased than when it can spread its own poison to others, without advantaging itself. *P.* If it be thus, let no one suffer his barber to come near him, to mow down his beard; or, for safety, let every man shave himself. *G.* But if, by way of security, what if each of them were to keep his lips close, and not to speak a word till the barber has done, and is gone about his business? *P.* That won't do, because the infection may be taken in by the nostrils. *G.* I have a remedy for that. *P.* Pray what? *G.* To do as the chemists, wear a mask with glass-eyes that may let in the light, and a tube also that may be fastened behind from under the arms, by which the air may pass to and from the mouth and nostrils. *P.* This is well enough, if we had nothing to fear from a touch of his fingers, from his linen, or from his comb and scissars. *G.* The surest way then, I find, will be for every man to let his beard grow down to his knees. *P.* Now you are right; and let us have an arret to forbid every barber acting as a surgeon. *G.* At this rate you leave the barbers to starve. *P.* If they abate of their price, they may possibly have more work. *G.* Well, let it be so. *P.* The next thing we have to do, is to get another arret, forbidding two persons, under severe penalty, drinking out of the same cup. *G.* This will not go down in England. *P.* Another, that no two people lye together in the same bed, unless they be man and wife. *G.* Very good. *P.* Besides an edict, that no host or hostess suffer any stranger to lye in the same sheets where another has layen before. *G.* What will you do with the Germans, who neither wash nor shift themselves above twice in a year? *P.* Let them be more cleanly. Besides these, let the old custom be abolished of saluting with a kiss. *G.* What! although at church? *P.* Let every one set about the reform. *G.* But what think you? Can there be any danger in this way of confabulating? *P.* Let us avoid what Homer says, Ἄγχι χὼν κεφαλὴν. And on the other hand, let the hearer always keep his mouth shut. *G.* Twelve tables will scarce contain these laws."

The following papers show that our neighbours can cure what they call venereal diseases by acid vapours, and that they do not confine themselves to *chlorine.*

---

[From our Correspondent at Paris.]

*Eruptions occasioned by an unknown Venereal Affection.*

Miss ——, born at Soissons, of healthy parents, had attained her tenth year in the most flourishing state of health, while her beauty excited general admiration. A slight sore throat came on, which was the signal of a most deplorable scene.—Her gums were covered with aphthæ; soon after, a swelling of the glands of the neck, redness of the skin on the shoulders, eruptions all over the face and thighs, fall of the eye-lashes and eye-brows, the mouth turned aside, with frightful ugliness; but no general symptoms, except great leanness and dejection. In this state she was brought to the Hospital for Children.

M. Jadelot, the head physician, seeing no reason to think there was any particular cause for the complaint, treated the eruption, as he had done many others successfully, with sulphurous baths, but to no purpose; and the complaint went on increasing for two months, though recourse was had to all the remedies that seemed most suitable for it. The mother of the patient came to the hospital to take her away, and, being questioned on the supposed cause of her daughter's situation, she declared she could say nothing positive about it, that her other children were in good health, and that only one of them, who had formerly been in the army, complained on his return of pains in his limbs, and prickings in his throat; but that she could not think her daughter had caught his complaint, though she often drank out of his glass.

The next and following days, mercury was administered; after the fourth dose, the patient felt better, and continued so by the use of this medicine for six weeks, when her strength and spirits returned, and the redness of the shoulders had disappeared; but the eruption on the face and thighs, though less violent, still remained, and, finally, resisted mercury in every shape. It was a separate complaint, which required a direct treatment; the sulphurous baths were resumed, and, in less than fifteen days, a complete cure was obtained.

The particular conclusion to be drawn from this is, that sulphurous baths may cure venereal eruptions, and that, in a general way, it may be affirmed, that there are cases in which two distinct complaints (one of which is cause, and the other effect) may exist simultaneously; that each requires a different treatment; and, that the employment of this method should follow the order of succession of the two diseases.

PRUS, D.M.P.

---

### *A Note on Sulphurous Fumigations against obstinate and inveterate Venereal Eruptions.*

B——, fifty years old, a native of Wansert, living at Givet, a mariner by profession, born of robust parents, who died in the decrepitude of age; contracted, in the year 1810, a venereal complaint which resisted a course that does not seem to have been very judicious. The first means employed was bleeding in the arm; then frictions, which soon brought on a salivation, aphthæ in the throat, &c.; sudorific drinks, and other proper medicines, with two blisters in the arms,—all which produced no good effect.

The patient admits, that this failure should, perhaps, be attributed to his neglect in following the regimen prescribed for a whole year, and to some excesses in drink, to which his profession often exposed him.

However this might be, in 1811, his face, shoulders, and some other parts of his body, particularly the loins, became covered with moist eruptions, producing a whitish thick running. He was treated without success for two years by a Dutch physician, and his complaint grew worse. Disgusted at the inefficacy of his medicines, and, finding no benefit from a cautery in the right arm, and a seton in the neck, he confined himself to sudorific drinks and baths at home, which he took frequently. However, the eruption made frightful progress, and had already affected all the outside of the head, and made such ravages internally, that the left eye was soon entirely lost, and the right much threatened.

Such was the deplorable state of this patient when he came to me on the 16th of September, 1817. After having heard and examined him, I had no doubt that an inveterate and ill-treated pox was a principal cause, or at least a complication, of his eruptive disease;—I subjected him immediately to sulphurous fumigations, to dispose him for the internal treatment which the pox required.

After the first fumigations, he had a gastric embarrassment, weaknesses, general lassitude, and lost his appetite; but these symptoms soon yielded to the evacuations I prescribed. From that time, the fumigations were never interrupted.

The eruptions on the shoulders, back, and loins, began to remove first, and, on the second fumigation, those parts were entirely freed. Those on the surface of the head, which had destroyed the left eye, and greatly affected the right, together with those on the two arms, resisted longer; nevertheless, after the fortieth fumigation, the right eye was out of danger, and the skin grew white by degrees, and insensibly resumed its natural state.

The treatment of this patient was followed for two months, by Dr. Picouline, a privy-counsellor in Russia, and a man of distinguished learning. He was seen by Le Roux, dean of the faculty of Paris, and by Drs. Dupuytren, Bouillon-Lagrange, Demangeon, Tartra, &c. He was presented to them the 15th of last November, just two months from the beginning of the course, in a state of perfect health; the right eye quite sound, and his skin perfectly smooth and white, &c.

Thus, in the space of two months, by means of seventy-five fumigations, and the depurating syrup, with the addition of superoxygenated muriat, in the dose of sixteen grains to two pounds of syrup, (in all, eight pounds of syrup and sixty-four grains of muriat,) a venereal eruption disappeared, which, for seven years, had resisted every remedy which, till now, had been reckoned the best.

GALIS, D.M.P.

*Note of the Editor of the Gazette.*—I have examined this patient since his cure, and had the means of comparing his present state with what he was two months and a half ago. M. Galis, having had a drawing taken of him (which makes one shudder), it is very easy still to perceive the resemblance, from the enormous and numerous scars with which his body is all covered. I declare that I never saw so striking an instance of a cure, and this makes me often return to the subject of sulphurous fumigations, by which almost miraculous results are obtained, and precisely in cases in which all other remedies almost always fail.

---

Our motto must be our apology for this *farrago*, which the reader will only find introductory to the succeeding Analysis.—Edit.

